# ID 302 - Análise da Efetividade da Inteligência Artificial: tecnologia de dados em saúde para gestão do Sistema Único de Saúde

**DOI:** 10.5327/2237-9622.2025.v34s1.302

**Published:** 2025-11-25

**Authors:** Olga Veloso da Silva, Maria Helena Machado Vieira, Andrea Georgia de Souza Frossard, João Batista Lima do Nascimento, Anderson Veloso da Silva, Marcio de Vasconcelos Maciel

**Keywords:** efetividade, inteligência artificial, Sistema Único de Saúde, tecnologia em saúde, gestão em saúde, qualidade

## Abstract

**Introdução:**

O desenvolvimento das novas tecnologias em saúde trouxe para a humanidade o empoderamento e um ritmo acelerado por meio da inteligência artificial (IA) como ferramenta no diversificado ecossistema tecnológico. Na saúde, a IA tem potencial promissor de reorganização de todos os processos de gestão do Sistema Único de Saúde (SUS). Este estudo se justifica pelo caráter inovador da análise das tecnologias em saúde na saúde pública, e como relevante ferramenta e força motriz para o progresso. A IA pode ser aliada na efetividade para a gestão do SUS como ferramenta tecnológica, definindo a cultura organizacional das instituições públicas. Sendo assim, o objetivo geral deste estudo é: analisar, nos estudos empíricos, a efetividade do uso da IA na gestão do SUS; e os objetivos específicos são: identificar os estudos empíricos sobre o uso da IA na gestão do SUS; descrever e analisar o uso da IA no âmbito da saúde pública.

**Método:**

Trata-se de uma revisão integrativa que utilizou as cinco fases. Na etapa de formulação da pergunta, elaborou-se a seguinte questão: O que trazem os estudos empíricos sobre a inteligência artificial e a gestão do SUS? Na fase de estratégias de busca e coleta de dados, utilizaram-se as bases de dados: LILACS, SciELO, MEDLINE, Scopus e PubMed. Os termos utilizados como descritores foram: inteligência artificial, SUS, tecnologia em saúde, gestão em saúde e efetividade; e a palavra-chave foi: qualidade. Quanto aos critérios de inclusão, destacam-se: estudos com recorte temporal entre janeirode 2023 a agosto de 2024; idiomas: português, espanhol e inglês; estudos publicados no formato de artigos científicos. Os excludentes foram: estudos cujo cenário não fosse o Brasil; o idioma não estivesse na inclusão; e estudos do tipo anais, editoriais, comentários e opinião.

**Resultados:**

Na avaliação dos estudos incluídos, utilizaram-se as diretrizes da checagem PRISMA () dos artigos selecionados. Com isso, foram identificados 339 artigos, 112 para leitura, 42 elegíveis e 22 incluídos na revisão.

**Conclusão:**

Na fase de discussão dos dados, emergiram quatro categorias: "Os aspetos benéficos do uso da IA para a gestão do SUS": tem alto potencial de melhorar a qualidade de vida dos pacientes; a eficiência e a agilidade nos processos das instituições hospitalares; as ferramentas com a IA podem ser úteis para os pacientes se utilizadas de forma segura; automatiza diversas atividades; realiza vasta coleta e análise de dados. "Os aspectos negativos do uso da IA na gestão do SUS": falta de mão de obra qualificada e especialistas em IA; questões como o impacto no mercado de trabalho e a perda das relações humanas interpessoais; risco de perda da capacidade cognitiva, criatividade, pensamento crítico e intuição. "As ameaças que a IA oferece para o SUS": questões relacionadas à privacidade, ética e segurança das informações dos pacientes. "As oportunidades que a IA oferece ao SUS": capacitação para utilização dessa tecnologia; melhorar a qualidade do aprendizado institucional; com a IA, o conhecimento chega a lugares inacessíveis. Conclui-se que a IA surge como um diferencial tecnológico de dados com um poder de magnitude possibilitando o alcance e a impulsão para o SUS tornar-se dinâmico e com vasta possibilidade, que será o diferencial para a efetiva melhoria na qualidade da saúde pública.

